# The Changes in Expression of Na_V_1.7 and Na_V_1.8 and the Effects of the Inhalation of Their Blockers in Healthy and Ovalbumin-Sensitized Guinea Pig Airways

**DOI:** 10.3390/membranes11070511

**Published:** 2021-07-07

**Authors:** Michaela Kocmalova, Ivana Kazimierova, Jana Barborikova, Marta Joskova, Sona Franova, Martina Sutovska

**Affiliations:** 1 Biomedical Centre Martin, Jessenius Faculty of Medicine, Comenius University, 036 01 Martin, Slovakia; kocmalova@uniba.sk (M.K.); kazimierova@uniba.sk (I.K.); 2 Department of Pharmacology, Jessenius Faculty of Medicine, Comenius University, 036 01 Martin, Slovakia; jakubikova34@uniba.sk (J.B.); joskova@uniba.sk (M.J.); franova@uniba.sk (S.F.)

**Keywords:** cough, bronchial hyperreactivity, ciliary beating frequency, asthma therapy, Nav1.8 channels

## Abstract

Background: The presented study evaluated the suppositional changes in the airway expression of Nav1.8 and Nav1.7 and their role in the airway defense mechanisms in healthy animals and in an experimental asthma model. Methods: The effects of the blockers inhalation on the reactivity of guinea pig airways, number of citric-acid-induced coughs and ciliary beating frequency (CBF) were tested in vivo. Chronic inflammation simulating asthma was induced by repetitive exposure to ovalbumin. The expression of Nav1.7 and Nav1.8 was examined by ELISA. Results: The Nav 1.8 blocker showed complex antitussive and bronchodilatory effects and significantly regulated the CBF in healthy and sensitized animals. The Nav1.7 blockers significantly inhibited coughing and participated in CBF control in the ovalbumin-sensitized animals. The increased expression of the respective ion channels in the sensitized animals corresponded to changes in CBF regulation. The therapeutic potency of the Nav1.8 blocker was evidenced in combinations with classic bronchodilators. Conclusion: The allergic-inflammation-upregulated expression of Nav1.7 and Nav1.8 and corresponding effects of blocker inhalation on airway defense mechanisms, along with the Nav1.8 blocker’s compatibility with classic antiasthmatic drugs, bring novel possibilities for the treatment of various respiratory diseases. However, the influence of the Nav1.8 blocker on CBF requires further investigation.

## 1. Introduction

Respiratory tract receptors and defensive reflexes, such as sneezing and coughing, have been extensively studied. The physiological roles of these receptors in modifying the respiratory and cardiovascular systems are of great interest, as is their potential importance in respiratory diseases [1]. The airways and lungs are innervated by heterogeneous populations of vagally derived sensory neurons originated from nodose or jugular ganglia, whose differences in functional, neurochemical, neuroanatomical and molecular expression characteristics have been clearly demonstrated regarding the regulation of respiratory reflexes in guinea pigs [2,3,4,5].

The initiation of airway defense reflexes is driven by the activation of afferent nerve endings through various receptors and ion channels. The activation of voltage-gated sodium channels (Na_V_s) plays a key role in action potential (AP) generation and conduction in excitable cells, and their mutations or damage are responsible for the pathogenesis of various diseases [6]. There are nine distinct pore-forming alpha subunits of Na_V_s that come from nine different genes. These channels are referred to as Na_V_1.1–Na_V_1.9. With the exception of Na_V_1.4, which is expressed in striated muscle, and Na_V_1.5, which is expressed in cardiac myocytes, all the Na_V_1s are expressed in neurons [7]. Studies in guinea pigs have shown that the vagal neurons innervating the respiratory tract express the Na_V_1.7, Na_V_1.8 and Na_V_1.9 isoforms [6,8].

Na_V_s are classified according to their sensitivity to tetrodotoxin (TTX) [9]. TTX is a blocker that preferentially blocks Na_V_1.1–1.4, Na_V_1.6 and Na_V_1.7 (TTX-sensitive channels) with low nanomolar potency as well as short-lasting effects and is approximately 100–1000-fold less potent in blocking Na_V_1.5, Na_V_1.8 and Na_V_1.9 (TTX-resistant channels) [6].

Na_V_1.7 is highly involved in the generation of action potentials in various types of excitable cells. The knockdown of Na_V_1.7 expression in the vagal sensory ganglia of guinea pigs abolished AP conduction and significantly suppressed the coughing induced by citric acid (AC). Na_V_1.7 is also highly expressed on unmyelinated C-fibers that also contain the neuropeptide substance-P and mediate bronchoconstriction. Furthermore, Rice et al. [10] and Jo et al. [11] demonstrated that the sodium channel Na_V_1.7 is present extraneuronally, e.g., within skin vascular myocytes and the endothelium of arterioles [10] or on cultured human smooth muscle cells [12].

Extensive evidence indicates that coughing caused by airway inflammation is mediated by the capsaicin-sensitive vagal afferent C-fibers, which, in the guinea pig, are the C-fibers originating from vagal jugular ganglia [13]. Na_V_1.8 is expressed on airway sensory C-fibers and Aδ-fiber neurons, and PGE2 increases their conductivity, spreading excitement and total irritability. Na_V_1.8 is a TTX-resistant ion channel that is significantly different from other neuronal TTX-resistant Na_V_s in its fast voltage activation and inactivation dependence [12]. This allows Na_V_1.8 to participate in the spreading of the AP during the period of permanent depolarization [14]. One of Na_V_1.8′s features is the fact that its usually high threshold for activation is significantly reduced after the exposure of sensory neurons to inflammatory mediators.

This different distribution paves the way for developing selective Na_V_ blockers with therapeutic indexes much greater than can be obtained with nonselective local anesthetics [15]. Furthermore, the expression of Na_V_ can be influenced by inflammatory mediators, as reported by Strickland et al. [16], which showed changes in Na_V_1.7, Na_V_1.8 and Na_V_1.9 expression in a rat model of Freund’s complete adjuvant (FCA)-induced chronic inflammatory joint pain. The alteration of Na_V_1.7, Na_V_1.8 or Na_V_1.9 expression leads to distinct changes in sensitivity in either inflammatory or neuropathic pain models [17].

The currently available antitussive drugs often have only limited efficacy in inhibiting excessive coughing in acute and chronic inflammatory respiratory diseases. Postganglionic parasympathetic nerves are responsible for reflex parasympathetic bronchospasm and mucus secretion. The mucus secretion may contribute to pathological coughing in some cases; likewise, cholinergic bronchoconstriction may increase cough sensitivity in those who suffer from hypersensitive cough syndromes [7]. Little information is available regarding their role (if any) in airway smooth muscle [18]. 

The specific expression, function and increasing evidence of the upregulation of the previously mentioned subtypes Na_V_1.7 and Na_V_1.8 by several mediators of allergic inflammation led to the assumption that selective blockers of these subtypes may represent a novel therapeutic strategy for diseases and symptoms associated with neuronal hyperexcitability in the respiratory tract. Except from their ability to inhibit coughing, these potential drugs can exhibit bronchodilatory activity. The object of many discussions is their cilio-modulatory activity, but this has not been confirmed yet.

The goal of the current study was to generate new knowledge about the pharmacodynamic effects of the Na_V_ blockers in the respiratory tract. Their influence on the parameters of airway defense mechanisms after single-dose administration were examined under physiological conditions and during the inflammation of the airways. In our experimental conditions, we tested their ability to relieve coughing, decrease specific airway resistance (sRaw) (airway reactivity in in vivo conditions) and influence the ciliary beating frequency (CBF), one of the main parameters determining the efficiency of mucociliary clearance. Given the fact that several studies confirmed Na_V_ upregulation during inflammatory conditions, we compared the changes in the expression of various subtypes of Na_V_ between healthy and sensitized groups of animals. One of the goals of the current study was to determine the most effective substance regarding the previously mentioned parameters and test its efficacy against that of clinically used antiasthmatics.

## 2. Materials and Methods

Adult male TRIK strain guinea pigs weighing 150–350 g (obtained from The Department of Experimental Pharmacology, Slovak Academy of Sciences, Dobra Voda, Slovakia) were used in the present study. All the protocols described in the study were approved by the local Ethic Committee (IRB00005636, decision No. EK 40/2018) and followed the aims and objectives of the ARRIVE guidelines. The investigation followed the Guide for the Care and Use of Laboratory Animals: Eighth Edition (2010) published by the US Committee for the Update of the Guide for the Care and Use of Laboratory Animals; the National Research Council; the EU-adopted Directive 2010/63/EU of the European Parliament and of the council on the protection of animals used for experimental and other scientific purposes; and Slovak law regulating animal experiments. To ensure the stability of the laboratory conditions (ambient temperature of 21–24 °C and relative humidity of 55 ± 10%) during the performance testing, an LG multi-type air-conditioner (LG Neo plasma, Slovakia) was used.

The research was complied with the commonly accepted ‘3Rs’. A total of 105 animals were used in the presented study. The animals were divided into 15 groups, each consisting of 7 animals (Table 1). Experimental airway inflammation was induced in 13 groups. The groups were divided into control groups—2 negative and 2 positive—and 11 experimental groups: 9 groups used for testing several concentrations of the investigated substances and 2 groups used to prove the effects of Na_V_1.8 blocker combinations with clinically used bronchodilatory antiasthmatics. The negative groups (sensitized OVA+ and unsensitized OVA−) received isotonic saline (0.9% NaCl, 5 min inhalation), and the positive control groups, salbutamol (4 × 10^−3^ mol/L, 5 min inhalation) or codeine phosphate (10 mg/kg; per os). ProTx III (10^−8^, 10^−7^ or 10^−6^ mol/L and 5 min inhalation), huwentoxin IV (10^−9^, 10^−8^ or 10^−7^ mol/L and 5 min inhalation), A803467 (10^−8^, 10^−7^ or 10^−6^ mol/L and 5 min inhalation) were administered to 9 experimental groups. Two groups underwent treatment with a combination of A803467 at 10^−8^ mol/L with salbutamol at 4.10^−3^ mols/L or A803467 at 10^−8^ mol/L with ipratropium at 10^−3^ mol/L.

Tetrodotoxin (TTX) served for comparative purposes only in in vitro tests. All the chemicals used for the in vitro experiments were also administered directly on biological samples. For CBF testing in in vitro conditions, the same concentrations used for the inhalations were used. TTX was applied at concentrations of 10^−9^–10^−7^ mol/L.

ProTx III is a potent Na_V_1.7 blocker (IC50 = 2.5 nM). It also inhibits Na_V_1.1, Na_V_1.2, Na_V_1.3 and Na_V_1.6 in the nanomolar range. Huwentoxin IV preferentially inhibits neuronal Na_V_1.7, 1.2 and 1.3 (the IC50 values are 26, 150 and 338 nM, respectively), compared to the muscle subtypes Na_V_1.4 and 1.5 (IC50 ≥ 10 μM). The small molecule A803467 is a selective blocker of Na_V_1.8 channels (the IC50 value is 8 nM). A-803467 is over 100-fold more potent against Nav1.8 than human Na_V_1.2, 1.3, 1.5 and 1.7. For experimental purposes, the IC50s of the tested drugs were used. For the dose-dependence test, 1-fold higher and 1-fold lower concentrations were used.

### 2.1. Chemicals

Salbutamol, ovalbumin from chicken egg white, histamine and DMSO solution were purchased from Sigma Aldrich (St. Louis, MO, USA). ProTx III, A803467, huwentoxin IV and TTX were obtained from TOCRIS (Bristol, UK). All the other chemicals used were purchased as listed: codeine phosphate (Slovakofarma Hlohovec, Slovakia); sodium chloride solution and methacholine (ApliChem, Darmstadt, Germany); RPMI 1640 medium (Invitrogen/Gibco, USA); aluminum hydroxide (CentralChem, Bratislava, Slovakia); and citric acid (AC) (ACROSorganics, Bratislava, Slovakia). According to the manufacturers’ instructions, the chemicals were dissolved in water for injection (salbutamol, ProTx III, huwentoxin IV and codeine), saline (ovalbumin, methacholine, AC, aluminum hydroxide and histamine), 10% DMSO (A803467) and acidic buffer (TTX).

### 2.2. Model of Experimentally Induced Airway Inflammation

An experimental model of allergic airway inflammation was induced by the 21-day administration of an allergen—ovalbumin (OVA)—to the adult male guinea pig strain TRIK. OVA, adsorbed on aluminum hydroxide (Al(OH)_3_, was applied in a repetitive parenteral manner at the same dose (5 mg of OVA and 100 mg of Al(OH)_3_—1st day i.p. and s.c., 4th day i.p. and 9th day s.c. The allergen was also administered by inhalation on the 12th, 15th, 18th and 20th days using a double-chamber bodyplethysmograph box for small animals (HSE type 855, Hugo Sachs Elektronik, Germany). All tests with the sensitized animals were performed 24 h after the last allergen administration.

### 2.3. Cough Reflex Assessment

The cough reflex was evoked by the inhalation of 0.3 M AC. An AC aerosol created by a nebulizer (PARI jet nebulizer, Paul Ritzau, Pari-Werk GmbH, Germany; output, 5 L·s^−1^; particle mass median diameter, 1.2 µm) was delivered into a nasal chamber during a 3-min interval. Two trained observers counted the number of cough efforts according to the presence of typical movement, cough sounds and airflow curve changes. The cough response was measured prior to the administration of any agent (baseline measurement, N value in graphs) and then 60 and 240 min after its application according to ERS guidelines [19].

### 2.4. Airway Smooth Muscle Reactivity In Vivo

The specific airway resistance (sRaw) was expressed as the airway reactivity in in vivo conditions. Awake guinea pigs were placed into a double-chamber bodyplethysmograph box for small animals (Hugo Sachs Elektronik, type 855) composed of separate nasal and body chambers. The sRaw values were calculated according to Pennock [20]. The airway resistance was recorded for 1 min immediately after histamine (10^−6^ mols/L) and methacholine (10^−6^ mols/L) inhalation before (N in graphs) and 60 and 240 min after the single administration of the substances. There was also a 1 min time interval between each bronchoconstrictor agent when the fresh air was blown into the nasal chamber.

### 2.5. Ciliary Beating Frequency

A PeCon Temp Controller 2000-2 (PeCon GmbH, Erbach, Germany) was utilized for the control and maintenance of the nutritive medium for the cilia temperature (RPMI 1640 Medium) and microscopic glass slides (37–38 degrees C). A sample of ciliary epithelium was obtained from the trachea using the brushing method in experimental in vitro conditions from negative control groups (OVA−, OVA+). A cytology brush (2.5-mm diameter) was placed in saline before brushing and then inserted directly into the trachea, rotated gently to collect ciliated cells and moved back. The cilia were suspended in 1 mL of RPMI 1640 Medium and used to make a microscopic preparation. The solution of tested and control drugs was applied directly on ciliary cells collected from trachea. An inverted phase contrast microscope (Zeiss Axio Vert. A1; Carl Zeiss AG, Göttingen, Germany) was used to examine the biological specimens. Impaired ciliated cells were excluded from this experiment. Over 15 min, with time series analysis, sequential 10-s video files were recorded at 1-min intervals using a digital high-speed video camera (Basler A504kc; Basler AG, Germany) at a frame rate ranging from 256 to 512 fps (frames per second). The video records were analyzed by virtual instrumentation using the Ciliary Analysis software (LabVIEW™) to generate a ciliary region of interest (ROI). This measurement method is based on a frequency analysis of intensity variance curve with the implementation of a fast Fourier transform algorithm [13]. Potential artefacts were filtered by the comparison of the ROI with the relevant video recording. The CBF was selected as the kinematic parameter for describing ciliary movement in the airways.

### 2.6. Enzyme-Linked Immunosorbent Assay (ELISA)

Homogenates from the bronchus and whole lung tissue were prepared by sonication (2 min) with a power output of 700 W (homogenizer, Stuart SHM2, ECOMED, Žilina) according to the manufacturer’s instructions. For analysis, the supernatant was collected into sterile tubes to determine the expression of Na_V_1.7 (after two freeze–thaw cycles to further break the cell membranes) and Na_V_1.8 (immediately after homogenization). The expression of the Na_V_1.7 and Na_V_1.8 isoforms was determined in lung and bronchial homogenates using a commercial ELISA kit for Guinea Pig Na_V_1.7, ELISA kit #MBS745877 (MyBioSource, USA), and Sodium Channel Protein Type 10 Subunit Alpha (SCN10A), ELISA Kit #MBS9349047 (MyBioSource, USA). The absorbance at 450 nm was measured using the Varioskan^®^ Flash version 2.4.5.

### 2.7. Statistical Analysis

All the obtained data are shown as means ± the standard error of the mean (S.E.M.). For their statistical analysis, t-tests and ANOVA with Bonferroni post-hoc tests were used, as appropriate. The results with *p* < 0.05 and lower were considered statistically significant.

## 3. Results

The aim was to verify the role of the voltage-gated sodium channels in the respiratory tract and also the differences between physiological and pathological situations in the airways. The changes of all tested parameters between OVA− and OVA+ groups are obvious. The recorded increase in sRaw values between OVA− and OVA+ group is almost 3 times higher and the number of coughs efforts grew almost by 50%. Substances capable of blocking Na_V_1.7 (huwentoxin IV and ProTx III) and Na_V_1.8 (A803467) were applied to animals by single-dose inhalation, and all the parameters in vivo were measured before and 60 and 240 min after drug inhalation. Their effects on the AC-induced cough reflex and ability to influence the sRaw elicited by the administration of bronchoconstrictors (histamine and methacholine) were monitored. The ability to reduce the CBF after the local application of the tested substances was monitored under in vitro conditions.

### 3.1. Results

#### 3.1.1. Effect on Cough Reflex

**Figure 1:**
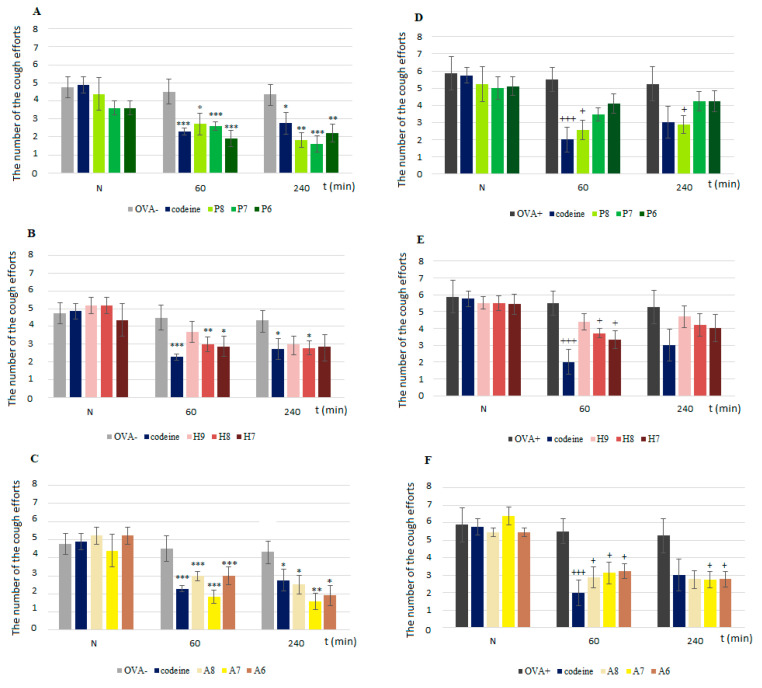
The number of cough efforts after administration of Nav 1.7 and Nav 1.8 blockers. N—measurement before any drug administration; 60 and 240—minutes after drug administration; (**A**)—ProTx III in physiological conditions; (**B**)—huwentoxin IV in physiological conditions; (**C**)—A803467 in physiological conditions; (**D**)—ProTx III in pathological conditions; (**E**)—huwentoxin IV in pathological conditions; (**F**)—A803467 in pathological conditions. P8—inhalation of ProTx III at concentration of 10^−8^ mols/L; P7—inhalation of 10^−7^ mols/L; P6—10^−6^ mols/L; H9—inhalation of huwentoxin IV at concentration of 10^−9^ mols/L; H8—10^−8^ mols/L; H7—10^−7^ mols/L; A8—inhalation of A803467 at concentration of 10^−8^ mols/L; A7—10^−7^ mols/L; A6—10^−6^ mols/L. Number of animals for each group: 7. Left column presents data obtained under physiological conditions. Right column presents data obtained after development of allergic inflammation. * *p* < 0.05, ** *p* < 0.01 and *** *p* < 0.001 vs. OVA–; + *p* < 0.05 and +++ *p* < 0.001 vs. OVA+.

Under physiological conditions, ProTx III (the selective Na_V_1.7 blocker) reduced the number of coughs at all tested doses (Figure 1A). After the development of airway inflammation, ProTx III decreased the number of coughs with statistical significance only when the measurement was performed 60 min after the inhalation of the agent at a concentration of 10^−6^ mols/L (Figure 1D). In healthy animals, huwentoxin IV, with a preferential effect on Na_V_1.7, significantly reduced the number of cough efforts, especially for the middle tested concentration, at which the cough suppression lasted longer (Figure 1B), and also in OVA-sensitized guinea pigs, where the suppressive effect was limited to the measurement 60 min after the drug inhalation (Figure 1E). The effect of A803647 was noticeable in both groups of animals, unsensitized (Figure 1C) and sensitized (Figure 1F), and for all the tested drug concentrations. The effect of A803467 was also similar to that of the control antitussive drug codeine.

#### 3.1.2. Influence of Na_V_1.7 and Na_V_1.8 Blockers on sRaw Values

**Figure 2:**
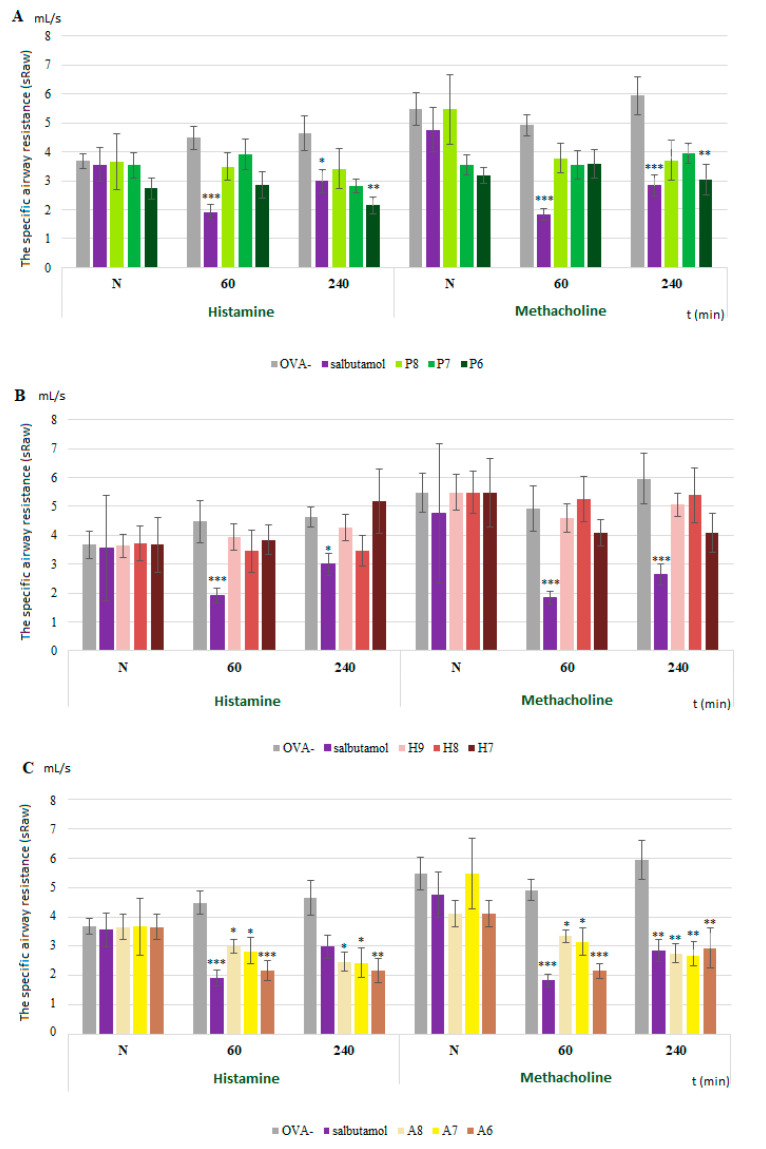
The changes in specific airway resistance values (sRaw) after administration of Na_V_1.7 and Na_V_1.8 blockers in physiological conditions. sRaw values are expressed as means ± S.E.M. N—measurement before drug administration; 60 and 240—minutes after drug administration; (**A**) of Figure 2—ProTx III; (**B**)—huwentoxin IV; (**C**)—A803467. For more detailed explanation, see the legend of Figure 1. * *p* < 0.05, ** *p* < 0.01 and *** *p* < 0.001 vs. OVA−.

**Figure 3:**
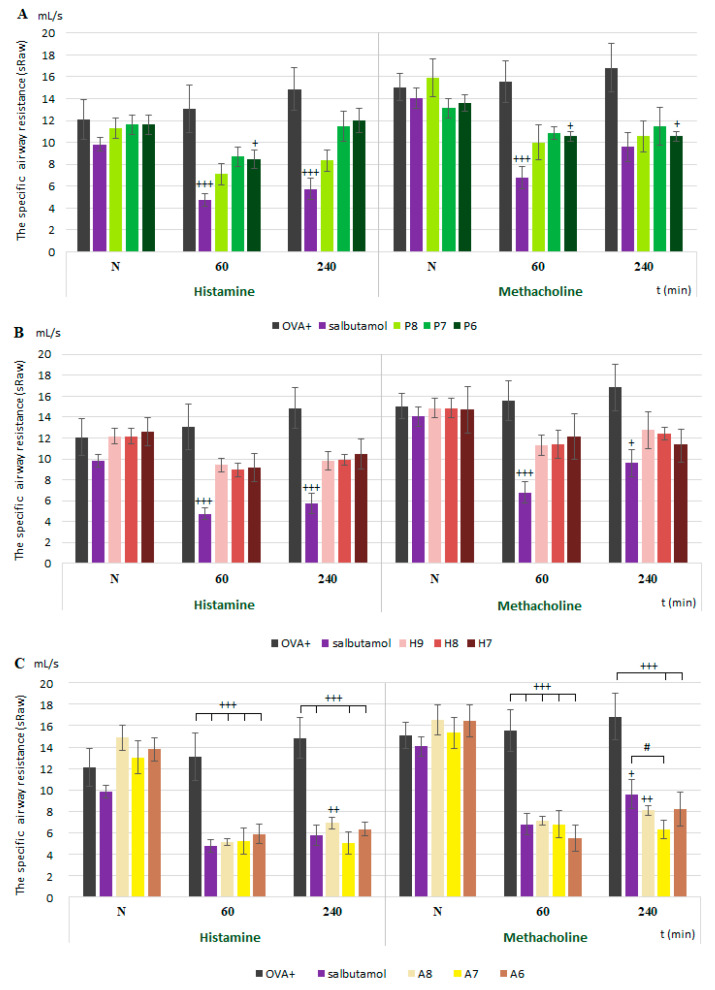
The changes in specific airway resistance values (sRaw) after administration of Na_V_1.7 and Na_V_1.8 blockers in allergic conditions. sRaw values are expressed as means ± S.E.M. sRaw values are expressed as averages ± SDs. N—measurement before drug administration; 60 and 240—minutes after drug administration; (**A**) of Figure 3—ProTx III; (**B**)—huwentoxin IV; (**C**)—A803467. For further detailed explanation, see legend of Figure 1. + *p* < 0.05, ++ *p* < 0.01 and +++ *p* < 0.001 vs. OVA+.

The value of sRaw was recorded for 2 min after the inhalation of each bronchoconstrictor (histamine and methacholine) (Figure 2 and Figure 3). ProTx III exhibited an effect on the sRaw values only for the highest tested concentration (Figure 2A). Huwentoxin IV did not significantly influence the sRaw values (Figure 2B). Under physiological conditions, we recorded the following results: A803467 significantly reduced sRaw values, without a difference between the tested bronchoconstrictors (Figure 2C).

The pharmacodynamic effect of Nav1.7 as well as Nav1.8 blockers did not change during the development of inflammation (Figure 3). ProTx III (Figure 3A) significantly relieved the bronchoconstriction after histamine or metacholine inhalation but only at the highest concentration. Huwentoxin IV (Figure 3B) did not reduce the sRaw values after histamine or metacholine inhalation. A803467 reduced the sRaw values regardless of the bronchoconstrictor tested (Figure 3C) and, moreover, had an even more significant effect, according to some measurements, than the clinically used bronchodilator salbutamol.

#### 3.1.3. Ciliary Beating Frequency Results

**Figure 4:**
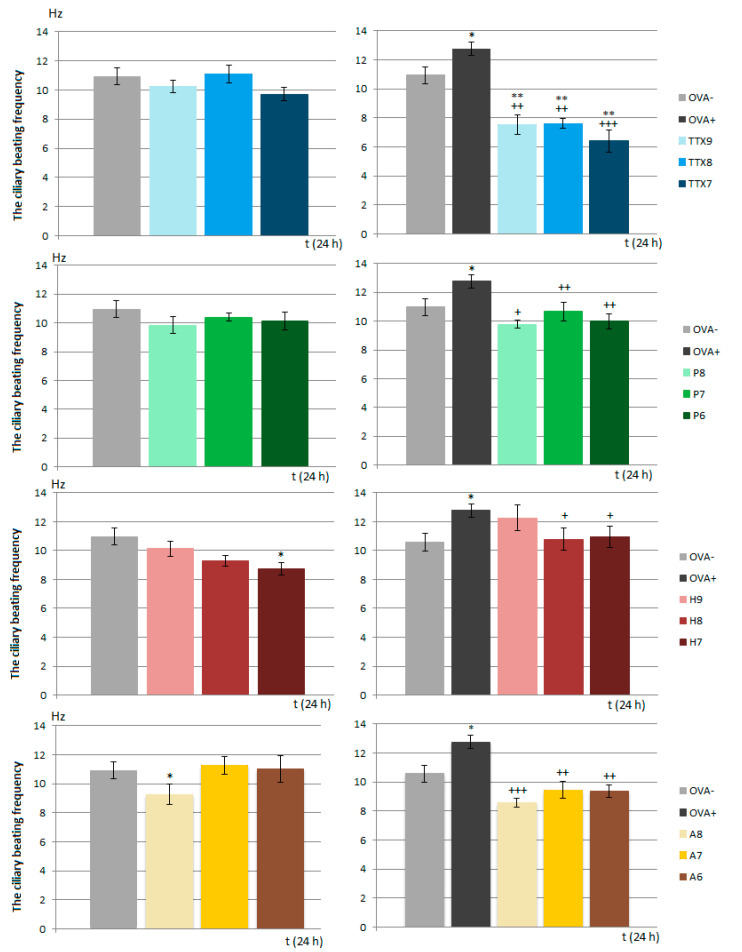
The values of ciliary beating frequency expressed in Hz, measured in unsensitized (OVA−) and sensitized (OVA+) animals after incubation of ciliated cells with individual concentrations of tested substances. P—ProTx III; H—huwentoxin IV; A—A803467. For a detailed explanation, see the legend of Figure 1. Left column presents data obtained under physiological conditions. Right column presents data obtained after development of allergic inflammation. * *p* < 0.05 and ** *p* < 0.01 vs. OVA−; + *p* < 0.05, ++ *p* < 0.01 and +++ *p* < 0.001 vs. OVA+.

As expected (Figure 4), the CBF values were significantly increased in the group of animals that underwent ovalbumin sensitization.

Generally, TTX and Na_V_1.7 and Na_V_1.8 blockers modulated the CBF but had relatively little effect when tested under physiological conditions: TTX and ProTx III did not significantly influence ciliary beating, Huwentoxin IV significantly decreased the CBF only at the highest concentration used. The IC50 of the Na_V_1.8 blocker A803467 significantly inhibited the CBF, the main parameter determining mucociliary clearance; however, higher doses did not have a similar impact on cilia movements. Higher doses of A803467 increased the CBF but without statistical significance.

Under the OVA-induced inflammatory conditions, the CBF was significantly and dose-dependently suppressed by the non-selective Na_V_ channel blocker TTX. Furthermore, the frequency of cilia movements was significantly reduced compared to that in the OVA− group. All the tested concentrations of ProTx III and A803467 as well as two higher concentrations of huwentoxin IV significantly decreased the CBF in the control group, which underwent sensitization by OVA, to the level of the CBF values in the healthy control group.

#### 3.1.4. Changes in Na_V_1.7 and Na_V_1.8 Expression during Development of Inflammation

**Figure 5:**
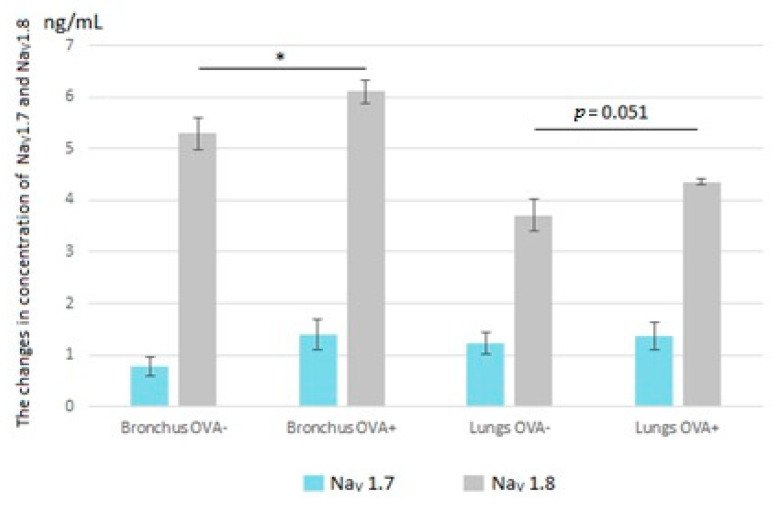
Changes in expression of isoforms Na_V_1.7 and Na_V_1.8 before (OVA−) and after development of chronic allergic inflammation (OVA+). Number of animals for each group: 7; * *p* < 0.05 vs. OVA−.

Based on the knowledge regarding the neuronal localization of TTX-sensitive and TTX-resistant Na_V_ channels, we sought to determine whether the Na_V_1.7 and 1.8 subtypes were present in bronchial and lung tissue homogenates and if there were changes between the healthy group of animals and animals with allergic inflammation. This hypothesis was verified by the sandwich ELISA method. Isolated tissue of the respiratory tract (bronchial or pulmonary) was positive for Na_V_1.7 and Na_V_1.8 expression, unambiguously in favor of the Na_V_1.8 isoform, in both physiological and pathophysiological conditions (Figure 5). The expression of Na_V_1.8 in the bronchial homogenate of the healthy group was significantly lower than that in the group with ongoing chronic inflammation. These changes in lung tissue only narrowly exceeded statistical significance (Figure 5).

#### 3.1.5. Na_V_1.8 Blocker as Potential Asthma Reliever in Clinical Practice

Nav1.7 reduced the cough reflex and ciliary beating frequency but the physiological role, expression increase and constant effect on airway reactivity confirmed to us the decision to investigate only the effect of the Nav1.8 blocker in combination. Because of previously mentioned data and from the clinical practice point of view, we focused attention on the Na_V_1.8 blocker. We tested combinations of A803467 with salbutamol and ipratropium, clinically used relievers of asthma. We observed that such combinations affected the number of cough efforts, sRaw values and CBF.

**Figure 6:**
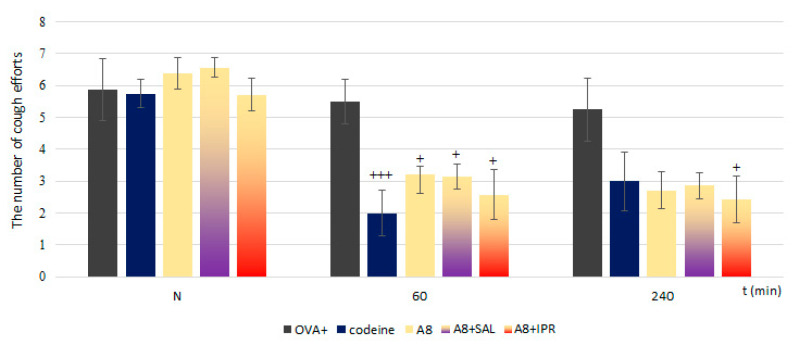
The number of cough efforts after administration of A803467 with salbutamol/ipratropium combinations in pathological conditions. N—the number of coughs before drug administration; 60/240—minutes after drug administration; A—A803467 concentration of 10^−8^ mols/L; A + S—A803467 + salbutamol concentration of 4 × 10^−3^ mols/L; A + I—A803467 + ipratropium concentration of 10^−3^ M/L. Number of animals for each group: 7; + *p* < 0.05 and +++ *p* < 0.001 vs. OVA+.

Figure 6 presents the effects on AC-provoked coughing. Both combinations exhibited antitussive effects similar to A803467 itself, lower than those of the opioid antitussive codeine. A803467 and ipratropium were more effective in cough suppression; however, the differences between individual groups were not statistically significant.

**Figure 7:**
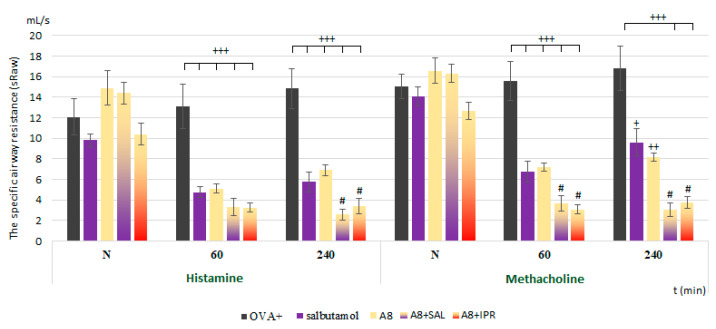
The changes in specific airway resistance values (sRaw) after the administration of the Na_V_1.8 blocker alone and with salbutamol/ipratropium in chronic inflammatory conditions. sRaw values are expressed as means ± S.E.M, and N means measurement before any drug administration; 60 and 240 are the minutes after the administration of a particular drug; A—A803467 concentration of 10^−8^ mols/L; A + S—A803467 + salbutamol concentration of 4 × 10^−3^ mols/L; A + I—A803467 + ipratropium concentration of 10^−3^ mols/L. Number of animals for each group: 7; + *p* < 0.05, ++ *p* < 0.01 and +++ *p* < 0.001 vs. OVA+. # *p* < 0.05 vs. salbutamol.

The effects of combinations on the sRaw were synergic (Figure 7). However, the Na_V_1.8 blocker alone significantly decreased the sRaw values regardless of the bronchoconstrictor used, but combinations with clinically used antiasthmatics intensified this effect. Both combinations tested were significantly more effective in the suppression of histamine- and methacholine-induced airway constriction than the classic bronchodilatory drug salbutamol.

**Figure 8:**
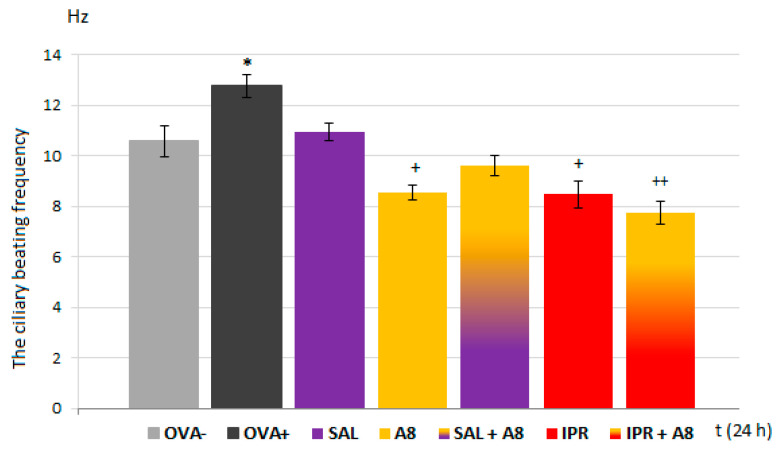
The values of ciliary beating frequency (Hz), measured in unsensitized (OVA−) and sensitized (OVA+) animals after the incubation of ciliated cells with A803467, classic bronchodilatory drugs and their combinations in OVA+ groups as follows: SAL—incubation with salbutamol concentration of 4 × 10^−3^ mols/L; A8—A803467 at concentration of 10^−8^ mols/L alone; SAL + A8—A803467 with salbutamol; IPR—incubation with ipratropium at concentration of 10^−3^ mols/L; IPR + A—A803467 with ipratropium. * *p* < 0.05 vs. OVA−; + *p* < 0.05 and ++ *p* < 0.01 vs. OVA+.

The effects of salbutamol, ipratropium and their combinations with the Na_V_1.8 blocker on the CBF were also observed (Figure 8). Salbutamol had no impact on the ciliary frequency. The antimuscarinic drug ipratropium led to a decrease in this parameter, similar to A803467. However, their combination had a synergic effect on ciliary movement and further suppressed this parameter. On the other hand, the combination of A803467 and ipratropium decreased the CBF less than A803467 alone, and this decline was not statistically significant.

## 4. Discussion

In the present study, we proved the role of Na_V_1.7 and Na_V_1.8 in the modulation of airway defense reflexes in physiological conditions as well as during the development of allergic inflammation. Na_V_1.7 and Na_V_1.8 blockers seemed to be effective cough relievers regardless of whether the cough reflex was elicited under physiological conditions or by the chemical stimulation of inflamed airways. Furthermore, the Na_V_1.8 blocker A803467 significantly reduced the sRaw, highlighting its bronchodilatory effect. We also proved the role of Na_V_ channels in the regulation of ciliary movement. The effect of Na_V_1.7 blockers on the CBF in physiological conditions, however, was minimal. By contrast, the lowest concentration of Na_V_1.8 tested significantly decreased the CBF. In pathological conditions, we proved the involvement of Na_V_1.7, especially in the TTX-mediated reduction of CBF, corresponding to the expression data. The obtained data significantly support the clinical use of the Na_V_1.8 blocker and its combination with clinically used antiasthmatics.

Coughing is the most important airway reflex that protects the airways and lungs from aspirated, inhaled irritants and accumulated secretions. Na_V_1.7 and Na_V_1.8 have been shown to be expressed in lung-specific vagal sensory neurons in human and animal models that display cough reflexes, e.g., guinea pigs [12], or on jugular and nodose ganglia [21]. These channels are upregulated in response to inflammatory mediators that are known to increase cough sensitivity [22]. Moreover, former and recent studies have already shown that cultured bronchial smooth muscle cells, including those from human airways, express Na^+^ currents [23]. Thus, sodium channels should be one of the several reasons for hyperreactivity, airway obstruction and pathological coughing.

The presence of Na_V_1.7 and Na_V_1.8 in homogenates from the tracheal and pulmonary tissue of healthy and OVA-sensitized guinea pigs was confirmed in this study. At the same time, we showed the involvement of Na_V_1.7 and Na_V_1.8 in the regulation of the cough reflex. Our experiments are consistent with numerous published findings [24,25]. Evans et al. [25] proved the involvement of Na_V_1.7 in the cough reflex, which explained benzonatate (a clinically used antitussive)’s mechanism of action in catecholamine A differentiated (CAD) cells, which express TTX-sensitive Na_V_ channels, composed primarily of Na_V_1.7 with smaller contributions of Na_V_1.1 and Na_V_1.3. The selective inhibition of Na_V_1.7 via gene silencing in nodose sensory neurons reduces the excitability of these neurons and inhibits mechanically induced and citric-acid-induced coughing in guinea pigs [26]. Muroi and Undem [15] describe the upregulation of Na_V_1.8 expression and significantly reduced threshold for activation after the exposure of sensory neurons to an inflammatory mediator. Brozmanova et al., in 2019 [13], proved that the Na_V_1.8 inhibitor A803467 reduced capsaicin-induced coughing at doses that did not affect the respiratory rate. This could be achieved by either the systemic or local (inhalation) administration of the drug. However, the clinical relevance of such consistent results originated from various experimental studies is still limited. The main reason is controversial data from clinical trials. In clinical trials using nebulized lidocaine, about 50% of patients reported successful cough suppression but more than 40% reported some side effects including dysphonia, oropharyngeal numbness and a bitter taste [27]. Furthermore, the results for a novel selective blocker targeting Na_V_1.7 (GSK2339345) that was investigated in a randomized, double-blind, placebo-controlled crossover study in 16 patients with refractory chronic cough suggested no antitussive effect [28].

The exaggerated ASM contractile response characterizing bronchial hyperresponsiveness is the main pathophysiologic factor involved in the development of reversible airway obstruction, e.g., that seen in asthma [29]. Since the airway constriction in patients with asthma is believed to be mediated by the vagus nerve, blockers of Na_V_ channels may be able to prevent airway constriction [30]. Na_V_ modulators are discussed more as reliable tussigens or antitussives in the literature, but their ability to control airway smooth contractility is still not exactly clear. Although there have been published reports partially proving the ability of Na_V_ channel blockers to reduce airway responsiveness to bronchoprovoking agents [31,32,33], each study has several limitations, e.g., the routes of Na_V_ blocker administration, the low channel selectivity of the tested compounds or the low effective doses used. Unlike nodose and jugular ganglia or the neurons, where both channels were found to be expressed equally [7,21], our findings show dominant Na_V_1.8 expression in whole tissue homogenates. This highlights their potential extra-neuronal presence, e.g., on airway smooth muscle. The blockers of Na_V_1.7 and Na_V_1.8 tested in our study were administered in a localized manner, by inhalation at different concentrations that were selected in order to avoid activating another channels. The sRaw measurements show a dominant role of Na_V_1.8 in the regulation of the airway reactivity/hyperreactivity induced by histamine and methacholine. These findings also correspond with the results for the expression. However, a more important role of Na_V_1.7 in the control of parasympathetic airway contraction in humans and guinea pigs was previously suggested according to its expression in parasympathetic ganglia and the inhibitory effects of TTX and a Na_V_1.7 blocker on the contraction of isolated tracheal smooth muscle [31]. These differences could be explained by the different methods for testing airway reactivity. Moreover, this study was focused on the regulation of airway reactivity under physiological conditions only. In the OVA-sensitized guinea pigs, the blockade of Na_V_1.8 efficiently reduced the bronchoconstriction induced by mediators involved in the hyperreactivity associated with the chronic inflammation of the airways. The standard of care for treating mild to moderate asthma has remained largely unchanged for many years. The Global Initiative for Asthma (GINA) recommends a stepwise approach for the treatment of asthma. The preferred inhalation treatment options for persistent asthma varies depending on the severity but, at standard, includes inhaled glucocorticoids and bronchodilators, e.g., β2-agonists or anticholinergics [33]. One of the main problems in the management of asthmatic patients, regardless of the severity, type and therapeutic strategy chosen, is the limited duration of full therapeutic responses (due to the downregulation of β2 receptors) or spectrum of side effects (anticholinergics). A Na_V_1.8 blocker was proved to be a compatible treatment in combinations with the β2 agonist salbutamol and anticholinergic drug ipratropium. This combination not only resulted in sufficient control but also had a synergic effect in decreasing the sRaw, the main parameter determining airway hyperresponsiveness.

Na_V_1.7 and Na_V_1.8 are believed to be preferentially distributed in peripheral sensory neurons and the C-fibers of neurons innervating airways [11,12]. Inflammatory mediators (e.g., amines, prostanoids, kinins and nerve growth factor) can reduce the thresholds for the activation of these channels [34] or increase their expression in sensory neuronal cell bodies [17]. It corresponds with our experiments that were also focused on changes in the presence of Na_V_1.7 and Na_V_1.8 in inflamed airways. Despite the ELISA showing increased expression of both of the studied channel isoforms in the tracheal and lung tissue homogenates, only Na_V_1.8 was significantly overexpressed in tracheal tissue. However, Na_V_1.8 overexpression in the lungs can be partly associated with initial remodeling changes and the proliferation of vascular myocytes, where the presence of Na_V_1.8 has already been proved [10,11].

Mucociliary clearance ensures that the airways remain free of inhaled particles and detritus. A synchronous and fast ciliary beat, as well as the right amount and consistency of mucous, is essential for functioning mucociliary clearance. CBF is a key parameter controlling the rate of mucociliary clearance, and it is believed to be under vagal control [35]. The presence of Na_V_1.7 and Na_V_1.8 on afferent fibers of the vagal nerve points to their possible involvement in the control of ciliary movements. A regulatory role of Nav ion channels in the tracheal CBF has already been suggested [32]; however, the information about the role of Na_V_ in ciliary beating remains limited. Our results significantly expand the knowledge related to the regulation of respiratory epithelium cilia motility. First, the incubation of ciliated epithelial cells with increased concentrations of the Na_V_1.8 blocker, which mimicked the inhalatory administration of the compound, lead to a statistically significant decrease in the CBF in samples from both healthy and OVA-sensitized airways. TTX and Na_V_1.7 blockers, tested under the same conditions, significantly reduced the CBF in OVA-sensitized animals. These findings confirm a role for Na_V_1.8 channels in the control of cilia movements and point to a novel inflammation-induced regulatory role of Na_V_1.7 in CBF modulation. However, the TTX-induced inhibition of the CBF was, in fact, up to 40% greater. This could be explained by the possible involvement of other TTX-sensitive Na_V_ channels in the regulation of airway cilia function. Ciliary ion channels are topics of active investigation. In the airways, cilia function in concert with airway mucus to mediate the critical function of mucociliary clearance, cleansing the airways of inhaled particles and pathogens. The treatment of respiratory diseases should not negatively affect the function of mucociliary clearance. The fact that the Na_V_1.8 blocker decreased ciliary beating in OVA-sensitized animals cannot be overlooked, despite the decline being to a level similar to physiological CBF. Significantly, the inhibitory effect of the Na_V_1.8 blocker was antagonized in combination with the β2 agonist salbutamol.

## 5. Conclusions

The presented work highlights new findings regarding Na_V_ involvement in airway defense mechanisms. Blockers of Na_V_1.7 and Na_V_1.8 administered by inhalation exerted reliable suppressive effects on the cough reflex experimentally elicited under physiological conditions or by the chemical stimulation of inflamed airways. Na_V_1.7 seems to be more important in the cough reflex than in the process of airway smooth muscle contraction/relaxation. The changes in respiratory tract physiology induced by inflammatory mediators highlight the fact that Na_V_1.7′s involvement might be more important in chronic inflammatory disorders of the airways. Na_V_1.8 modulators maintained their effects in the regulation of airway defense mechanisms under physiological conditions as well as after the development of inflammation. The importance of the mentioned Na_V_ subtype also confirms findings from the expression analysis confirming dominance in airway tissue presence and overexpression in bronchial homogenates after ovalbumin sensitization. Therefore, modulators of Na_V_ channels could be regarded as future treatments for respiratory diseases associated with pathological coughing and/or airway hyperreactivity. Their impact on the regulation of mucociliary clearance, especially the CBF, cannot be overlooked. Na_V_1.8 showed compatibility with the asthma relievers used the most often in the clinic. Their additive effects on sRaw seem to be relevant from a clinical medicine point of view.

## Figures and Tables

**Table 1:** Design of the study.

Group	The Name in the Graphs	Description of the Treatment	Number of Animals in the Group
OVA− saline	OVA−	5 min inhalation of 0.9% NaCl	7
OVA+ saline	OVA+	5 min inhalation of 0.9% NaCl after OVA	7
Codeine	codeine	per oral administration, dose 10 mg/kg before and after OVA	7
Salbutamol	salbutamol	5 min inhalation of 4.10^−3^ mol/L before and after OVA	7
A803467 10^−8^	A8	5 min inhalation of 10^−8^ mol/L before and after OVA	7
A803467 10^−7^	A7	5 min inhalation of 10^−7^ mol/L before and after OVA	7
A803467 10^−6^	A6	5 min inhalation of 10^−6^ mol/L before and after OVA	7
ProTx III 10^−8^	P8	5 min inhalation of 10^−8^ mol/L before and after OVA	7
ProTx III 10^−7^	P7	5 min inhalation of 10^−7^ mol/L before and after OVA	7
ProTx III 10^−6^	P6	5 min inhalation of 10^−6^ mol/L before and after OVA	7
Huwentoxin IV 10^−9^	H9	5 min inhalation of 10^−9^ mol/L before and after OVA	7
Huwentoxin IV 10^−8^	H8	5 min inhalation of 10^−8^ mol/L before and after OVA	7
Huwentoxin IV 10^−7^	H7	5 min inhalation of 10^−7^ mol/L before and after OVA	7
A803467 10^−8^ + salbutamol	A8 + SAL	5 min inhalation of 10^−8^ A8 and 4.10^−3^ mol/L SAL after OVA	7
A803467 10^−8^ + ipratropium	A8 + IPR	5 min inhalation of 10^−8^ A8 and 10^−5^ mol/L IPR after OVA	7
The total number of animals	105		

## Data Availability

The data that support the findings of this study are available on request from the corresponding author. The data are not publicly available due to them containing information that could compromise the privacy of the research participants.
